# Supplementary material for: Recent Advances in Biosensors for Diagnosis of Autoimmune Diseases
Source: Sensors (Basel). 2024 Feb 26;24(5):1510. doi: 10.3390/s24051510 (PMC10934086; doi:10.3390/s24051510)
Supplement: Supplementary file 1 [file sensors-24-01510-s001.zip › sensors-2838158-supplementary.pdf]

Review

Recent Advances in Biosensors for Diagnosis of Autoimmune Diseases

Ahlem Teniou [1], Amina Rhouati [1] and Jean-Louis Marty [2,*]

[1] Bioengineering Laboratory, Higher National School of Biotechnology, Constantine 25100, Algeria
[2] UFR Sciences, Universitéde Perpignan Via Domitia, 66860 Perpignan, France
* Correspondence: jlmarty@univ-perp.fr (J.-L.M.)

Supplementary Material

Citation: Teniou, A.; Rhouati, A.; Marty, J.-L. Recent Advances in Biosensors for Diagnosis of Autoimmune Diseases. *Sensors* **2024**, *24*, x. https://doi.org/10.3390/xxxxx

Academic Editor(s): Name

Received: date
Revised: date
Accepted: date
Published: date

Copyright: © 2024 by the authors. Submitted for possible open access publication under the terms and conditions of the Creative Commons Attribution (CC BY) license (https://creativecommons.org/licenses/by/4.0/).

Table S1: Comparison between the reported biosensor technologies for the diagnosis and monitoring of autoimmune diseases.

Method of detection	Target	Limit of detection	Linear range	Real samples	Refs
**Celiac disease**					
Electrochemical (CV)	TGA	1.8 ng/mL	0.005 - 1 µg/mL	Serum	[1]
Electrochemical (LSV)	TGA	260 ng/mL	0.26 - 6.9 µg/mL	Serum	[2]
Electrochemical (SWV)	DGPA	/	0.033 - 1.0 µg/mL	Serum	[3]
Electrochemical (CV)	TGA	0.72 U/mL	0.25 - 8.54 U/mL	Serum	[4]
Electrochemical (Chronoamperometry)	TGA IgA	4.6 U/mL	0 - 100 U/mL	Artificial human plasma	[5]
Electrochemical (DPV)	TGA	2.4 U/mL	3 - 100 U/mL	serum	[6]
Electrochemical (EIS)	AGA	46 ng/mL	0 - 1 µg/mL	Serum	[7]
Electrochemical (Amperometry) + Optical (SPR)	AGA	33 ng/mL	0 - 10 µg/mL	Serum	[8]
Piezoelectric (QCM)	TGA	1300 ng/mL	1300 -12,000 ng/mL	Serum	[9]
Electrochemical (CV)	TGA IgA<br>TGA IgG<br>AGA IgA<br>AGA IgG	2.45 U/mL<br>2.95 U/mL<br>3.16 U/mL<br>2.82 U/mL	0 - 100 U/mL		[10]
Electrochemical (DPV)	TGA	1 U/mL	3 - 40 U/mL	Serum	[11]
Electrochemical (Chronoamperometry)	TGA	2 U/mL	3 – 100 U/mL	Serum	[12]
Electrochemical (CV) + Optical (ECL)	TGA	0.5 ng/mL	1.5 ng/mL - 10 µg/mL	Serum	[13]
Optical (SPR)	TGA	/	30–3000 nM	Serum	[14]
Electrochemical (Amperometry) + IoT	TGA	3.2 AU/mL (IgA)<br>1.4 AU/mL (IgG)	0 - 30 AU/mL	Serum	[15]
**Multiple sclerosis**					
Electrochemical (Amperometry)	Anti-MBP	0.016 ng/mL	0.05 - 50 ng/mL	Serum	[16]
Electrochemical (EIS)	Osteopontin	0.98 fg/mL	0.05 - 10,000 pg/mL	Serum	[17]
	Anti-IL 12 antibodies	5 pg/mL	0 - 100 pg/mL	Serum	[18]
	anti-IL-12 antibodies	3.5 pg/mL	0.1 - 500 pg/mL	Fetal Bovine Serum	[19]
	Anti-MBO	0.1495 ng/mL	0.4875 - 2500 ng/mL	Serum and CSF	[20]
	Anti-MBP	0.18 ng/mL	/	Serum	[21]
Electrochemical (DPV)	MBP, Tau	0.3 nM (MBP) 0.15 nM (Tau proteins)	58 - 227 nM (MBP), 0.5 - 15.1 nM (Tau protein)	Blood sample and/or CSF	[22]
Fluorescence	miR-145	0.016 nM	0.1 nM - 1.6 nM	Serum	[23]

spectrophotometer					
Electrochemical (OFET)	MBP	1 ng/mL	1 - 500 ng/mL	/	[24]
Electrochemical (DPV)	miR-155	10 aM	10 aM – 1 μM	Serum	[25]
	BDNF	9 pg/mL	10 - 40 pg/mL	Fetal bovine serum	[26]
Optical (Lateral flow)	Osteopontin	0.1 ng/mL	10 - 500 ng/mL	Serum	[27]
**Rheumatoid arthritis**					
Optical (ECL)	Anti-CCP	0.2 pg/mL	0.001 - 15 ng/mL	Serum	[28]
Electrochemical (CV)	Anti-CCP	15 pg/mL	8 - 250 pg/mL	Serum	[29]
Electrochemical (EIS)	Anti-CCP	0.82 U/mL	1 - 800 U/mL	Serum	[30]
Optical (SERS)	Anti-CCP	0.18 U/mL	0–25 U/mL	Serum	[31]
Electrochemical (Amperometry)	IL-6	0.42 pg/mL	0.97 - 250 pg/mL	Serum	[32]
Optical (Colorimetry)	IgM-RF	4.15 IU/mL	/	Serum	[33]
Electrochemical (EIS)	IgM-RF	0.22 IU/mL	1 - 200 IU/mL	Serum	[34]
Electrochemical (amperometry)	IgM-RF + Anti-CCP	0.8 IU/mL (RF) 2.5 IU/mL (CCPA)	/	Serum	[35]
Electrochemical (SWV)	Anti-CCP	0.16 IU/mL (in PBS) 0.22 IU/mL (in human serum)	0.25 – 1500 IU/mL	Serum	[36]
Electrochemical (SWV+CV)	Anti-CCP	/	0.125 - 2000 pg/mL	Serum	[37]
Electrochemical (EIS)	IL-6	0.33 pg/mL	1 pg/mL - 15 mg/mL	Serum	[38]
Electrochemical (DPV)	TNF-α	0.52 pg/mL	1 - 100 pg/mL	Serum	[39]
Electrochemical (EIS)	TNF-α	0.67 pg/mL (in PBS) 0.78 pg/mL (in human serum)	1 - 1000 pg/mL	Serum	[40]
Electrochemical (EIS)	TNF-α	60 pg/mL	500 pg/mL - 100 ng/mL	Serum	[41]
**Lupus erythematous systemic**					
Electrochemical (Amperometry)	BAFF and APRIL	0.33 pg/mL (BAFF) 16.4 pg/mL (APRIL)	1.1 - 100 pg/mL (BAFF) 0.05 - 20 ng/mL (APRIL)	Serum	[42]
Electrochemical (Amperometry)	BAFF and APRIL	0.08 ng/mL (BAFF) 0.06 ng/mL (APRIL)	0.24 - 120 ng/mL (BAFF) 0.19 - 25 ng/mL (APRIL)	Serum	[43]
Piezoelectric (QCM)	Anti-TRIM21 and anti-TROVE2 autoantibodies	0.01 U/mL (anti-TRIM21) 0.005 U/mL (anti-TROVE2)	0.32 - 7.17 U/mL (anti-TRIM21) 0.07 - 1.46 U/mL (anti-TROVE2)	Serum	[44]
Electrochemical (Amperometry)	anti-dsDNA	8 μg/mL	/	Serum	[45]

Abbreviations: TGA: transglutaminase-antibody; **DGPA:** antibody against deamidated gliadin peptides; **AGA:** anti-gliadin antibody; **QCM:** quartz crystal microbalance; **IgA:** immunoglobulin A; **IgG:** immunoglobulin G; **tTG:** tissue transglutaminase; **IoT:**

Internet of things; **Anti-MBP:** autoantibodies against myelin basic protein; **IL:** interleukin; **EIS:** Electrochemical impedance spectrometry; **CSF:** cerebrospinal fluid; **miR:** micro-RNA; **BDNF:** brain-derived neurotrophic factor**; Anti-CCP:** Anti-cyclic citrullinated peptide antibody; **ECL:** electrochemiluminescence; **RF**: Rheumatoid factor; **TNF-α:** tumor necrosis factor alpha; **BAFF:** B-cell activation factor; **APRIL:** a proliferation-induced ligand; **Anti-dsDNA:** Anti-double stranded DNA; **CV:** cyclic voltammetry; **LSV:** linear sweep voltammetry; **OFET:** Organic Field Effect Transistor; **DPV:** differential pulse voltammetry; **SWV:** Square wave voltammetry; **SPR:** Surface plasmon resonance; **SERS:** surface-enhanced Raman scattering

References

1. Habtamu, H.B., et al., *Electrochemical Immunosensor Based on Nanoelectrode Ensembles for the Serological Analysis of IgG-type Tissue Transglutaminase.* Sensors, 2019. **19**(5): p. 1233.
2. Rosales-Rivera, L.C., et al., *Disulfide-modified antigen for detection of celiac disease-associated anti-tissue transglutaminase autoantibodies.* Analytical and Bioanalytical Chemistry, 2017. **409**(15): p. 3799-3806.
3. Puiu, M., et al., *A modular electrochemical peptide-based sensor for antibody detection.* Chemical Communications, 2014. **50**(64): p. 8962-8965.
4. Longo, S., et al., *Nanoelectrode ensemble immunosensor platform for the anodic detection of anti-tissue transglutaminase isotype IgA.* Journal of Electroanalytical Chemistry, 2022. **906**: p. 115984.
5. González-López, A., M.C. Blanco-López, and M.T. Fernández-Abedul, *Micropipette tip-based immunoassay with electrochemical detection of antitissue transglutaminase to diagnose celiac disease using staples and a paper-based platform.* ACS sensors, 2019. **4**(10): p. 2679-2687.
6. Martín-Yerga, D. and A. Costa-García, *Towards a blocking-free electrochemical immunosensing strategy for anti-transglutaminase antibodies using screen-printed electrodes.* Bioelectrochemistry, 2015. **105**: p. 88-94.
7. Rosales-Rivera, L., et al., *Electrochemical immunosensor detection of antigliadin antibodies from real human serum.* Biosensors and Bioelectronics, 2011. **26**(11): p. 4471-4476.
8. Wajs, E., N. Fernández, and A. Fragoso, *Supramolecular biosensors based on electropolymerised pyrrole–cyclodextrin modified surfaces for antibody detection.* Analyst, 2016. **141**(11): p. 3274-3279.
9. Manfredi, A., et al., *Piezoelectric immunosensor based on antibody recognition of immobilized open-tissue transglutaminase: An innovative perspective on diagnostic devices for celiac disease.* Sensors and Actuators B: Chemical, 2014. **201**: p. 300-307.
10. Neves, M.M.P.S., et al., *Multiplexed electrochemical immunosensor for detection of celiac disease serological markers.* Sensors and Actuators B: Chemical, 2013. **187**: p. 33-39.
11. Martín-Yerga, D., et al., *Enhanced detection of quantum dots by the magnetohydrodynamic effect for electrochemical biosensing.* Analyst, 2017. **142**(9): p. 1591-1600.
12. Nanni, P.I., et al., *Staple-Based Paper Electrochemical Platform for Celiac Disease Diagnosis.* ChemElectroChem, 2018. **5**(24): p. 4036-4045.
13. Habtamu, H.B., et al., *A sensitive electrochemiluminescence immunosensor for celiac disease diagnosis based on nanoelectrode ensembles.* Analytical chemistry, 2015. **87**(24): p. 12080-12087.
14. Cennamo, N., et al., *An innovative plastic optical fiber-based biosensor for new bio/applications. The case of celiac disease.* Sensors and Actuators B: Chemical, 2013. **176**: p. 1008-1014.
15. Giannetto, M., et al., *An integrated IoT-Wi-Fi board for remote data acquisition and sharing from innovative immunosensors. Case of study: Diagnosis of celiac disease.* Sensors and Actuators B: Chemical, 2018. **273**: p. 1395-1403.
16. Guerrero, S., et al., *Monitoring autoimmune diseases by bioelectrochemical detection of autoantibodies. Application to the determination of anti-myelin basic protein autoantibodies in serum of multiple sclerosis patients.* Talanta, 2022. **243**: p. 123304.
17. Zhou, S., et al., *Ti3C2Tx MXene and polyoxometalate nanohybrid embedded with polypyrrole: Ultra-sensitive platform for the detection of osteopontin.* Applied Surface Science, 2019. **498**: p. 143889.

18. La Belle, J.T., et al., *A cytokine immunosensor for multiple sclerosis detection based upon label-free electrochemical impedance spectroscopy.* Biosensors and Bioelectronics, 2007. **23**(3): p. 428-431.
19. Bhavsar, K., et al., *A cytokine immunosensor for Multiple Sclerosis detection based upon label-free electrochemical impedance spectroscopy using electroplated printed circuit board electrodes.* Biosensors and Bioelectronics, 2009. **25**(2): p. 506-509.
20. Derkus, B., et al., *Myelin basic protein immunosensor for multiple sclerosis detection based upon label-free electrochemical impedance spectroscopy.* Biosensors and Bioelectronics, 2013. **46**: p. 53-60.
21. Derkus, B., et al., *Alginate and alginate-titanium dioxide nanocomposite as electrode materials for anti-myelin basic protein immunosensing.* Sensors and Actuators B: Chemical, 2014. **192**: p. 294-302.
22. Derkus, B., et al., *Simultaneous quantification of Myelin Basic Protein and Tau proteins in cerebrospinal fluid and serum of Multiple Sclerosis patients using nanoimmunosensor.* Biosensors and Bioelectronics, 2017. **89**: p. 781-788.
23. Mansourian, N., M. Rahaie, and M. Hosseini, *A Nanobiosensor Based on Fluorescent DNA-Hosted Silver Nanocluster and HCR Amplification for Detection of MicroRNA Involved in Progression of Multiple Sclerosis.* Journal of Fluorescence, 2017. **27**(5): p. 1679-1685.
24. Song, J., et al., *Influence of Bioreceptor Layer Structure on Myelin Basic Protein Detection using Organic Field Effect Transistor-Based Biosensors.* Advanced Functional Materials, 2018. **28**(37): p. 1802605.
25. Shariati, S., A. Ghaffarinejad, and E. Omidinia, *Early detection of multiple sclerosis (MS) as a neurodegenerative disease using electrochemical nano-aptasensor.* Microchemical Journal, 2022. **178**: p. 107358.
26. Ayankojo, A.G., et al., *Electrochemical sensor based on molecularly imprinted polymer for rapid quantitative detection of brain-derived neurotrophic factor.* Sensors and Actuators B: Chemical, 2023. **397**: p. 134656.
27. Mukama, O., et al., *A highly sensitive and specific lateral flow aptasensor for the detection of human osteopontin.* Talanta, 2020. **210**: p. 120624.
28. Zhao, Y., et al., *Label-free ECL immunosensor for the early diagnosis of rheumatoid arthritis based on asymmetric heterogeneous polyaniline-gold nanomaterial.* Sensors and Actuators B: Chemical, 2018. **257**: p. 354-361.
29. Zhou, B., et al., *Immunosensing the rheumatoid arthritis biomarker through bifunctional aldehyde-amine linkers on an iron oxide nanoparticle seeded voltammetry sensor.* Nanomaterials and Nanotechnology, 2022. **12**: p. 18479804221085103.
30. Chinnadayyala, S.R. and S. Cho, *Electrochemical immunosensor for the early detection of rheumatoid arthritis biomarker: Anti-cyclic citrullinated peptide antibody in human serum based on avidin-biotin system.* Sensors, 2020. **21**(1): p. 124.
31. Chon, H., et al. *Highly sensitive immunoassay of anti-cyclic citrullinated peptide marker using surface-enhanced Raman scattering detection*. in *International Conference on Nano-Bio Sensing, Imaging, and Spectroscopy 2015*. 2015. SPIE.
32. Zhang, C., et al., *Microfluidic electrochemical magnetoimmunosensor for ultrasensitive detection of interleukin-6 based on hybrid of AuNPs and graphene.* Talanta, 2022. **240**: p. 123173.
33. Veigas, B., et al., *Antibody modified gold nanoparticles for fast colorimetric screening of rheumatoid arthritis.* Analyst, 2019. **144**(11): p. 3613-3619.
34. Chinnadayyala, S.R., et al., *Label-free electrochemical impedimetric immunosensor for sensitive detection of IgM rheumatoid factor in human serum.* Biosensors and Bioelectronics, 2019. **143**: p. 111642.

35. Guerrero, S., et al., *Electrochemical biosensor for the simultaneous determination of rheumatoid factor and anti-cyclic citrullinated peptide antibodies in human serum.* Analyst, 2020. **145**(13): p. 4680-4687.
36. Selvam, S.P., S.R. Chinnadayyala, and S. Cho, *Electrochemical nanobiosensor for early detection of rheumatoid arthritis biomarker: Anti-cyclic citrullinated peptide antibodies based on polyaniline (PANI)/MoS2-modified screen-printed electrode with PANI-Au nanomatrix-based signal amplification.* Sensors and Actuators B: Chemical, 2021. **333**: p. 129570.
37. Ma, J., et al., *Label-free Electrochemical Immunosensor for Sensitive Detection of Rheumatoid Arthritis Biomarker Anti-CCP-ab.* Electroanalysis, 2022. **34**(4): p. 761-771.
38. Tertiş, M., et al., *Label-free electrochemical aptasensor based on gold and polypyrrole nanoparticles for interleukin 6 detection.* Electrochimica Acta, 2017. **258**: p. 1208-1218.
39. Hosseini Ghalehno, M., M. Mirzaei, and M. Torkzadeh-Mahani, *Electrochemical aptasensor for tumor necrosis factor α using aptamer–antibody sandwich structure and cobalt hexacyanoferrate for signal amplification.* Journal of the Iranian Chemical Society, 2019. **16**(8): p. 1783-1791.
40. Yagati, A.K., M.-H. Lee, and J. Min, *Electrochemical immunosensor for highly sensitive and quantitative detection of tumor necrosis factor-α in human serum.* Bioelectrochemistry, 2018. **122**: p. 93-102.
41. Arya, S.K. and P. Estrela, *Electrochemical immunosensor for tumor necrosis factor-alpha detection in undiluted serum.* Methods, 2017. **116**: p. 125-131.
42. Arévalo, B., et al., *Simultaneous electrochemical immunosensing of relevant cytokines to diagnose and track cancer and autoimmune diseases.* Bioelectrochemistry, 2022. **146**: p. 108157.
43. Arévalo, B., et al., *Binary MoS2 nanostructures as nanocarriers for amplification in multiplexed electrochemical immunosensing: Simultaneous determination of B cell activation factor and proliferation-induced signal immunity-related cytokines.* Microchimica Acta, 2022. **189**(4): p. 143.
44. do Nascimento, N.M., et al., *Label-free piezoelectric biosensor for prognosis and diagnosis of Systemic Lupus Erythematosus.* Biosensors and Bioelectronics, 2017. **90**: p. 166-173.
45. Fagúndez, P., et al., *An electrochemical biosensor for rapid detection of anti-dsDNA antibodies in absolute scale.* Analyst, 2018. **143**(16): p. 3874-3882.
